# The correlation between multimodal radiomics and pathology about thermal ablation lesion of rabbit lung VX2 tumor

**DOI:** 10.3389/fonc.2022.941752

**Published:** 2022-07-28

**Authors:** Jin Chen, Yuan Yan, QingFeng Lin, Jian Chen, Jie Chen, ZhengYu Lin

**Affiliations:** ^1^ The Department of Interventional Radiology, First Affiliated Hospital of Fujian Medical University, Fujian Provincial Key Laboratory of Precision Medicine for Cancer, Fuzhou, China; ^2^ The Department of Interventional Radiology, Sanming Second Hospital, Sanming, China

**Keywords:** VX2 tumor, lung cancer, Thermal ablation, Magnetic Resonance Imaging, multimodal radiomics

## Abstract

**Objective:**

To explore the correlation of CT-MRI pathology with lung tumor ablation lesions by comparing CT, MRI, and pathological performance of rabbit lung VX2 tumor after thermal ablation.

**Methods:**

Thermal ablation including microwave ablation (MWA) and radiofrequency ablation (RFA) was carried out in 12 experimental rabbits with lung VX2 tumors under CT guidance. CT and MRI performance was observed immediately after ablation, and then the rabbits were killed and pathologically examined. The maximum diameter of tumors on CT before ablation, the central hypointense area on T2-weighted image (T2WI) after ablation, and the central hyperintense area on T1-weighted image (T1WI) after ablation and pathological necrosis were measured. Simultaneously, the maximum diameter of ground-glass opacity (GGO) around the lesion on CT after ablation, the surrounding hyperintense area on T2WI after ablation, the surrounding isointense area on T1WI after ablation, and the pathological ablation area were measured, and then the results were compared and analyzed.

**Results:**

Ablation zones showed GGO surrounding the original lesion on CT, with a central hypointense and peripheral hyperintense zone on T2WI as well as a central hyperintense and peripheral isointense zone on T1WI. There was statistical significance in the comparison of the maximum diameter of the tumor before ablation with a central hyperintense zone on T1WI after ablation and pathological necrosis. There was also statistical significance in the comparison of the maximum diameter of GGO around the lesion on CT with the surrounding hyperintense zone on T2WI and isointense on T1WI after ablation and pathological ablation zone. There was only one residual tumor abutting the vessel in the RFA group.

**Conclusions:**

MRI manifestations of thermal ablation of VX2 tumors in rabbit lungs have certain characteristics with a strong pathological association. CT combined with MRI multimodal radiomics is expected to provide an effective new method for clinical evaluation of the immediate efficacy of thermal ablation of lung tumors.

## Introduction

Lung cancer is the second most common malignant tumor in terms of morbidity and has the highest death rate in the world (1), and the lung is one of the most common metastatic target organs of malignant tumors. Lung metastases appear in 30%–40% of malignant tumor patients with advanced stage (2). Thermal ablation can effectively prolong the survival time of patients in the treatment of pulmonary malignant tumors, and the treatment efficacy of smaller lung tumors is close to the efficacy of surgery (3, 4). Currently, thermal ablation of lung tumors is mainly represented by radiofrequency and microwave. However, the lung tissue is poorer in heat conduction with the singleness post-ablation evaluation method, and the therapeutic efficacy is lower than that of liver tumor ablation. Therefore, the immediate efficacy assessment of post-ablation is extremely important.

This study aims to compare and analyze the multimodal radiomics, pathological manifestations, and the correlation between the acute thermal injury after radiofrequency ablation (RFA) and microwave ablation (MWA) in rabbit lung VX2 tumors, so as to provide more information for the precise evaluation of the efficacy of thermal ablation of lung tumors.

## Materials and methods

### Establishment of rabbit lung VX2 tumor model

Three New Zealand male rabbits (weight 2.0–2.5 kg) were inoculated with VX2 tumor strain (gifted by the First Affiliated Hospital of Fujian Medical University) after general anesthesia with ketamine 1 ml/kg and chlorpromazine 0.5 ml/kg. Tumors formed on the inner musculature of the hind legs of the rabbits. About 2–3 weeks after tumor formation, fish sarcoid tumor tissue with vigorous growth at the edge of the tumor block was excised, and the tumor tissue was cut into tumor blocks of about 1-mm^3^ size.

### Cultivation of tumor strains in rabbit lungs

Fifteen New Zealand male rabbits (weight 2.0–2.5 kg), were fasted for 12 h before the operation, their skin was prepared after general anesthesia, and they were fixed in a prone position on a CT (Somatom Emotion, Siemens) scan table. The appropriate puncture path was determined after the CT scan. After the rabbits were positioned, disinfected, and draped, a 17G coaxial needle was used to puncture the lower lung under CT guidance. Then the tissue block was inserted into the lung parenchyma through the coaxial needle. CT was reexamined to observe the growth of the tumor in the rabbit lung 2–4 weeks after implantation. When the tumor grew to about 10 mm in diameter, it was used for ablation.

**Figure 1:**
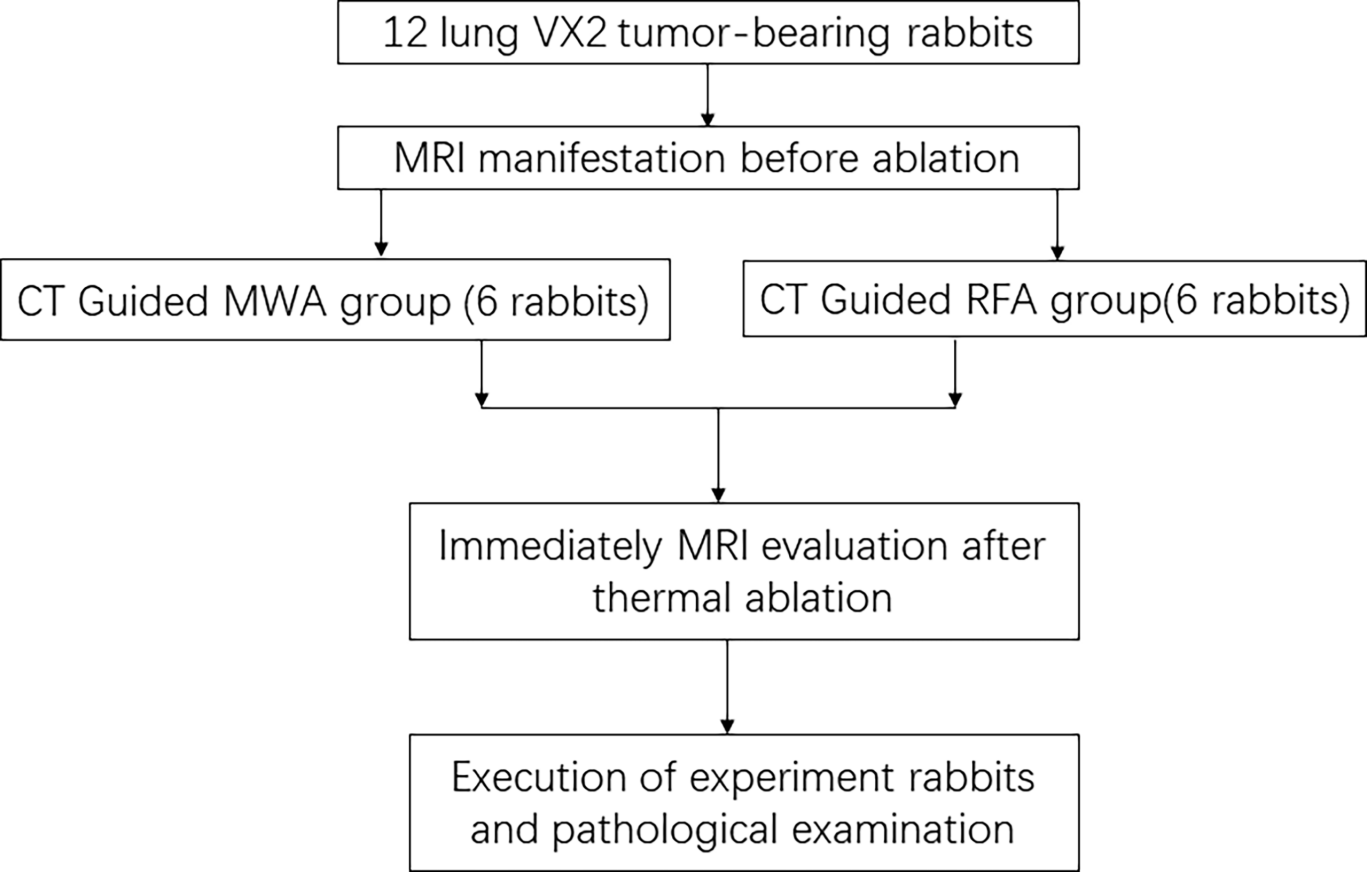
CONSORT flow diagram summarizes the process in this study. CONSORT, Consolidated Standards of Reporting Trials.

Thirteen experimental rabbits were successfully implanted with a VX2 tumor in the lung, showing a single nodule with soft tissue density in the lung by CT, and no tumor was found in 2 experimental rabbits. One tumor-bearing rabbit was killed and confirmed by pathological examination. There were 12 rabbits with VX2 tumors in the remaining lungs enrolled in our study (**Figure 1**). The tumor foci were located in the right lower lung in 6 rabbits and in the left lower lung in 6 rabbits. The average diameter of the tumor was 11.6 ± 2.9 mm (range 9.1–15.1 mm, median 11.3 mm).

### Pre-ablation MRI scan

After general anesthesia, 12 tumor-bearing rabbits underwent a 1.5-T MRI plain scan (Magnet Espree, Siemens, Germany) of the lung using the surface coil. The MRI scan sequence and parameters are shown in **Table 1**.

**Table 1:** MRI sequences and parameters.

Sequence	3D-VIBE-T1WI	FS-TSE-T2WI	DWI
Scan mode	Breath-free	Diaphragm trigger	Diaphragm trigger
TR	5.1 ms	4,000 ms	4,900 ms
TE	2.4 ms	80 ms	91 ms
FA	14°	90°	N/A
FOV	200 × 280 mm	200 × 210 mm	220 × 300 mm
NEX	1	2	2
Slice thickness	3 mm	3 mm	3 mm
Fat suppression	Yes	Yes	Yes
Gap	N/A	0.6 mm	0.6 mm
B value	N/A	N/A	50/800 mm^2^/s
Scan time	10–12 s	130–150 s	120–160 s

T1WI, T1-weighted image; T2WI, T2-weighted image; DWI, diffusion-weighted imaging; TR, repetition time; TE, echo time; FA, fractional anisotropy; FOV, field of view. VIBE, volumetric interpolated body examination; FS-TSE, fat saturation-turbo spin echo; NEX, number of excitation. N/A, not applicable.

### CT-guided thermal ablation of VX2 tumor in rabbit lung

Twelve tumor-forming rabbits were divided into 2 groups, 6 in each group. All tumor-bearing rabbits were placed in the prone position, and a CT scan was performed to select the appropriate puncture point. In the RFA group, a 17G needle with a 20-mm working radiofrequency electrode (star RF Electrode-Fixed, Starmed, Korea) with water cooling was gradually inserted under CT guidance. Through the water cooling cycle, the automatic pulse mode and the output power were set to 40–70 W; the continuous treatment time was 5–10 min.

In the MWA group, a microwave ablation antenna (15G, 150 mm, VISON Medical Equipment Co., Nanjing, China) with an outer diameter of 1.8 mm was gradually inserted into the distal end of the tumor under CT guidance, and the output power was set to 40–50 W with treating time of 4–6 min. The repeat CT scan showed that the surrounding ground-glass opacity (GGO) of the lesion exceeded 5–10 mm after ablation, which was considered complete ablation.

The needle was withdrawn after ablation, and a CT scan was performed again to observe whether there were complications such as pneumothorax, hemorrhage, and pleural effusion. If there was a pneumothorax and the lung was compressed by more than 20%, lung recruitment maneuver was performed by aspiration of the thoracic cavity.

All rabbit lung VX2 tumor ablation treatments were performed by physicians with more than 5 years of experience in tumor ablation.

### Post-ablation MRI scan

MRI was performed to observe the VX2 tumor in the rabbit lung immediately after ablation. The MRI sequences were the same as those before ablation. The experimental rabbits were killed and pathologically examined after an MRI scan.

### Statistical analysis

The maximum diameter of the tumor on CT before ablation, the central hypointense area on MRI T2-weighted image (T2WI) after ablation, the central hyperintense area on T1-weighted image (T1WI) after ablation, and the maximum diameter of coagulative necrosis on pathology were measured and compared. At the same time, the maximum diameter of the GGO around the tumor on CT after ablation, the peripheral hyperintense area on T2WI after ablation, the peripheral isointense area on T1WI after ablation, and the maximum diameter of the thermal injury area on pathology were also measured and compared. All indicators are expressed as mean ± SD. Statistical analysis was performed using the t-test of the SPSS software (version 22.0; IBM, Chicago, IL, USA), and p < 0.05 was considered statistically significant.

## Results

### MRI findings before ablation

**Figure 2:**
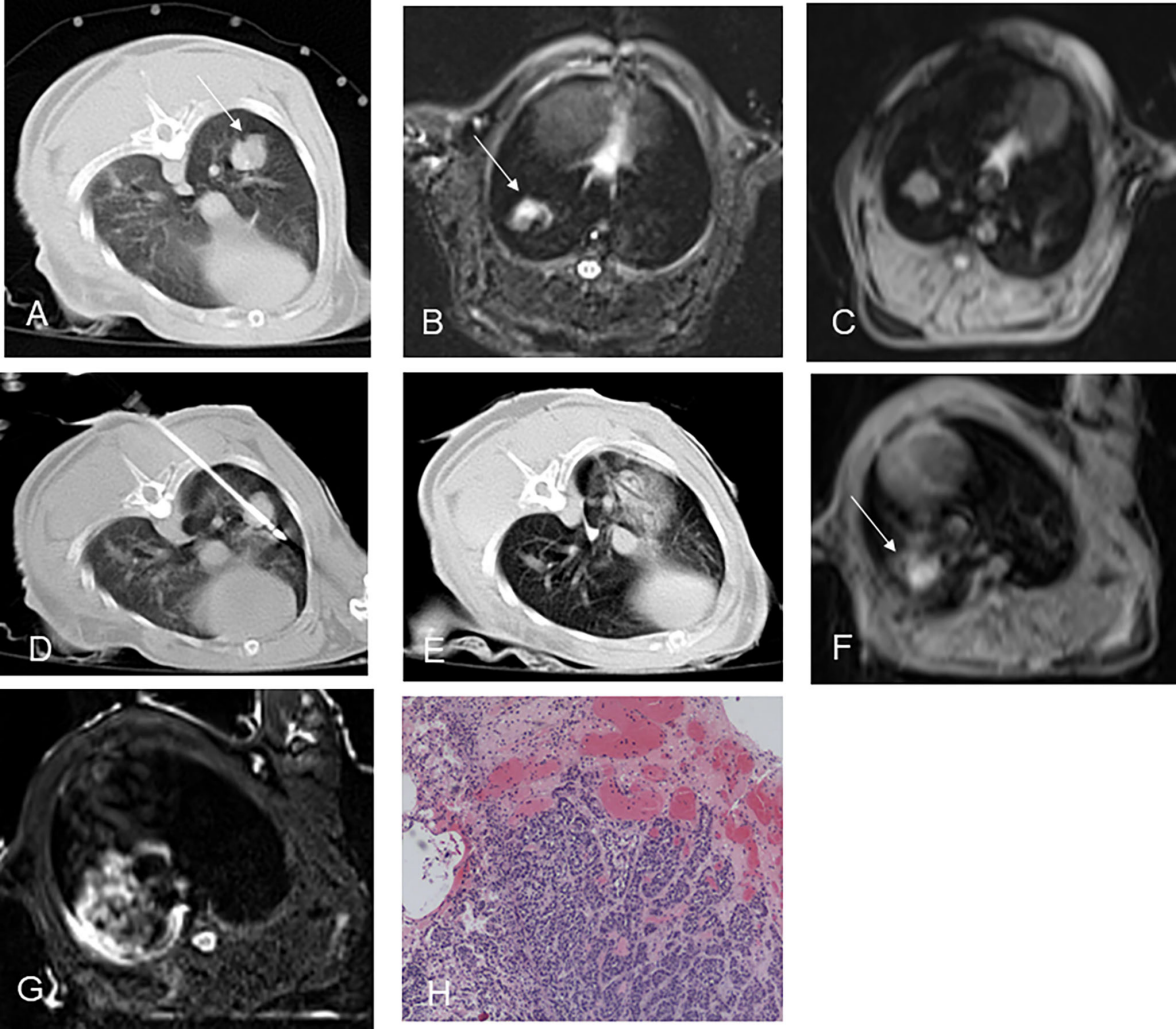
CT-guided RFA for rabbit lung VX2 tumor with MR and pathological findings. CT showed a VX2 tumor located in right lower lobe with 12 mm in diameter (**A**, arrow). The pre-ablation MR scan of the tumor showed hyperintensity on T2WI and isointensity on T1WI before RFA (**B, C**, arrow). Radiofrequency electrode was gradually punctured into the tumor under CT guidance **(D)**. GGO surrounding the lesion with 5 mm beyond was displayed immediately after RFA on CT **(E)**. Immediately after RFA, MR showed short T1 and short T2 signals in the central area and patchy iso-T1 and long T2 signals in the periphery (**F, G** arrow). Pathology showed coagulative necrosis of the ablation foci, with peripheral hemorrhage and inflammatory reaction zone **(H)**. RFA, radiofrequency ablation; T2WI, T2-weighted image; T1WI, T1-weighted image; GGO, ground-glass opacity.

**Figure 3:**
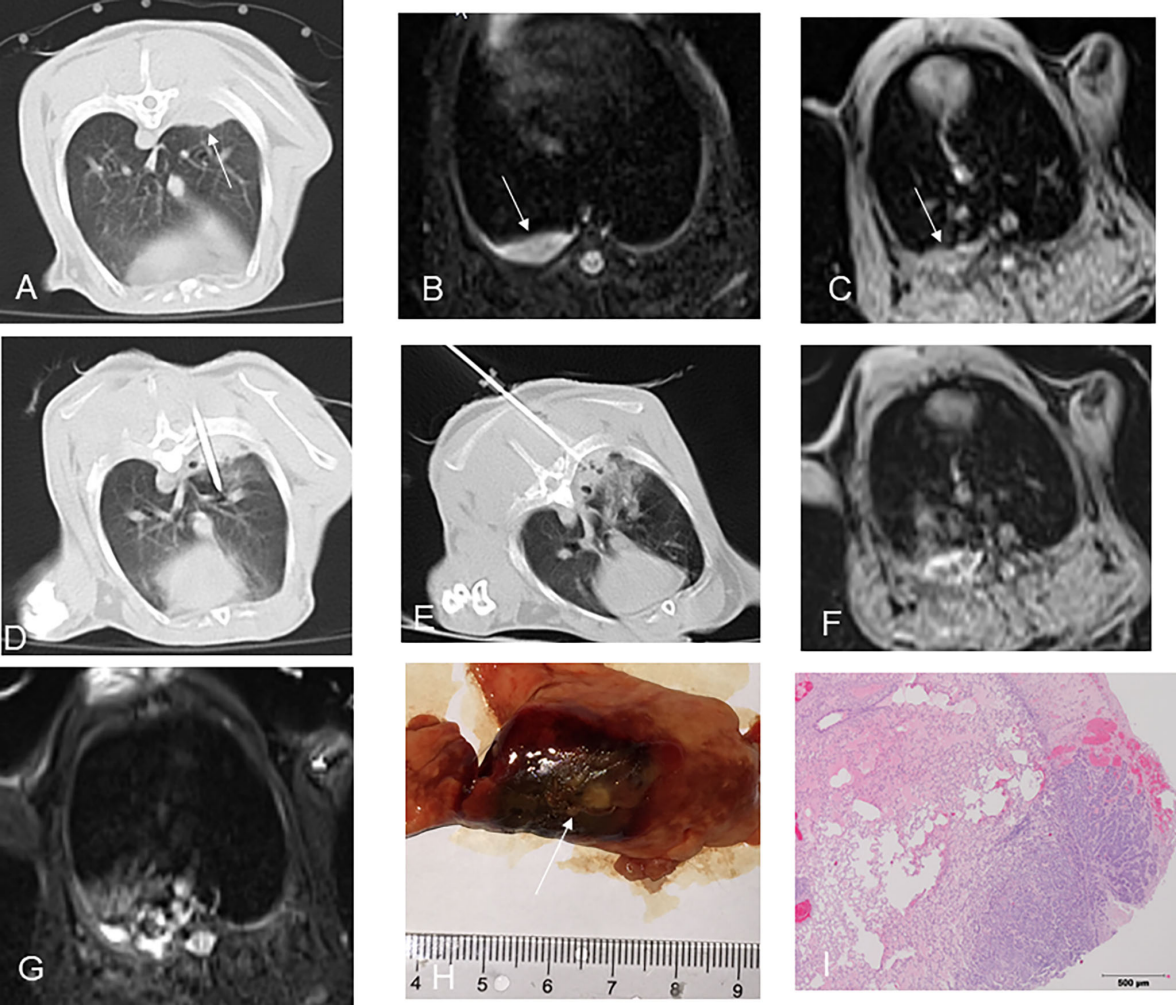
CT-guided MWA for rabbit lung VX2 tumor with MR and pathological findings. CT showed a VX2 tumor located in right lower lobe with 13 mm in diameter and invasion of adjacent pleura (**A**, arrow). The pre-ablation MR scan of the tumor showed hyperintensity on T2WI and isointense zone on T1WI before MWA (**B, C**, arrow). MW antenna was gradually punctured into the tumor under CT guidance **(D)**. GGO surrounding the lesion was displayed immediately after MWA on CT **(E)**. Immediately after MWA, MR showed short T1 and short T2 signals in the central area with patchy iso-T1 and long T2 signals in the periphery including adjacent pleura and chest wall **(F, G)**. Pathology showed coagulative necrosis of the ablation area including invasion of adjacent pleura, with peripheral hemorrhage and inflammatory reaction zone (**H, I** arrow). MWA, microwave ablation; T2WI, T2-weighted image; T1WI, T1-weighted image; GGO, ground-glass opacity. .

All rabbit lung VX2 tumors before ablation showed a round or slightly round-like hyperintense zone on T2WI and an isointense zone on T1WI compared with chest wall muscle (**Figures 2**, **3**), as well as a hyperintense zone on diffusion-weighted imaging (DWI) with clear boundaries.

### Procedure and CT/MRI findings after ablation

Thermal ablation was accomplished in all 12 rabbits’ lung VX2 tumors. CT showed GGO surrounding the original tumor in the lungs of 11 rabbits immediately after ablation (**Figure 2E**). In the remaining 1 rabbit, a blood vessel was injured during the RF electrode puncture into the target, resulting in patchy hemorrhage in the lung. The margin of the ablative zone was relatively unclear after the procedure.

In an MR scan immediately after ablation, the ablative areas showed a hypointense zone in the center surrounded by a ring-like hyperintense zone on T2WI. Meanwhile, the ablative areas showed a hyperintense zone in the center surrounded by a ring-like isointense zone on T1WI (**Figure 2F**). There was a hypointense zone in the center with a slightly ring-like hyperintense zone surrounding the ablated area, and the signal of original lesions was significantly reduced after ablation as observed on DWI.

### Pathology

The gross specimens of rabbit lung VX2 tumors after thermal ablation showed that the tumor tissues had a gray-white coagulation necrosis area after ablation, and the needle track area in the center of the ablation area was a fissure-like carbonization center, which was seen more in the MWA group than the RFA group. The lung tissue had a reddish-brown coagulation necrosis area, the coagulation necrosis areas were surrounded by a hemorrhagic zone, and a large area of congestion and edema was seen around the hemorrhage zone (**Figures 2H** and **3H**, **I**). One case showed residual VX2 tumor tissue in the RFA group confirmed by pathological examination. The remaining 11 cases showed complete ablation confirmed by pathological examination.

### Statistical results

There was statistical significance in the comparison of the maximum diameter of the tumor before ablation with a central hypointense zone on T2WI, a central hyperintense zone on T1WI after ablation, and pathological necrosis in both the RFA and MWA groups (p < 0.002, **Table 2**). There was no significant difference between the maximum diameter of the central hypointense zone on T2WI after ablation, the central hyperintense zone on T1WI after RFA, and the maximum diameter of coagulative necrosis on pathology in both the RFA and MWA groups (p > 0.05, **Tables 2**, **3**).

**Table 2:** Comparison of MD of tumor on CT pre-ablation, central hypointense zone on T2WI, central hyperintense zone on T1WI, and MD of coagulative necrosis on pathology post-ablation (mm).

	Pre-ablation (CT)	Central hypointense (T2)	Central hyperintense (T1)	Necrosis (pathology)
MD (RFA)	11.4 ± 3.2	13.6 ± 4.5	13.8 ± 6.3	15.0 ± 4.8
MD (MWA)	11.8 ± 2.9	14.2 ± 5.5	14.9 ± 3.3	15.2 ± 5.5

MD, maximum diameter; T2WI, T2-weighted image; T1WI, T1-weighted image; RFA, radiofrequency ablation; MWA, microwave ablation.

**Table 3:** Comparison of MD of MR performance and pathology after thermal ablation (mm).

	Central hypointense (T2) and central hyperintense (T1)	Necrosis (pathology)	p-Value
MD	14.5 ± 4.8	15.1 ± 2.8	0.07
	Peripheral hyperintense (T2) and peripheral isointense (T1)	Thermal injury (pathology)	
MD	23.8 ± 3.8	25.2 ± 6.3	0.004

MD, maximum diameter.

There was no significant difference between the maximum diameter of the peripheral hyperintense area on T2WI after ablation and the maximum diameter of the peripheral isointense area on T1WI after ablation (p > 0.05, **Table 4**). There was statistical significance in the comparison of the maximum diameter of GGO around the lesion on CT with a peripheral hyperintense zone on T2WI, peripheral isointense on T1WI after ablation, and thermal injury on pathology (p < 0.005, **Tables 3**, **4**).

**Table 4:** Comparison of MD of GGO around tumor on CT, peripheral hyperintense zone on T2WI, peripheral isointense on T1WI, and thermal injury on pathology post-ablation (mm).

	GGO (CT)	Peripheral hyperintense (T2)	Peripheral isointense (T1)	Thermal injury (pathology)
MD (RFA)	20.8 ± 4.5	23.7 ± 5.4	23.5 ± 6.2	24.9 ± 6.9
MD (MWA)	21.6 ± 6.5	24.2 ± 4.7	24.0 ± 3.8	25.2 ± 5.8

MD, maximum diameter; GGO, ground-glass opacity; T2WI, T2-weighted image; T1WI, T1-weighted image; RFA, radiofrequency ablation; MWA, microwave ablation.

## Discussion

At present, CT is commonly used to guide thermal ablation for lung tumors and evaluate the ablative efficacy immediately (3–5). GGO of the lung tissue covering the original tumor and exceeding 5–10 mm is usually considered a sign of successful ablation (6, 7). However, the GGO after ablation is sometimes confused with the hemorrhage of mechanical damage to the lung tissue during the puncture process, and the density of the tumors does not significantly change during the CT scan. Therefore, it is not accurate to use CT alone to evaluate the immediate effect of lung tumor ablation, especially when combined with postoperative puncture bleeding (8).

Miao et al. (9) performed RFA for rabbit VX2 lung tumor models and found that five typical isocenter areas formed around the radiofrequency needle after RFA: needle tract area, tumor coagulated necrosis area, lung parenchyma coagulated necrosis area, peripheral hemorrhage, and inflammatory area. In this study, the pathological changes of the rabbit lung VX2 tumor after thermal ablation were similar to those reported in the literature. MRI findings have certain characteristics after ablation due to MRI’s sensitivity to changes in tissue temperature and water content (10–12). Oyama (13) conducted a comparison study of MRI manifestations and pathology after RFA of normal pig lungs. After RFA of normal pig lungs, there was an inner zone of equal T1 short T2 signals with an outer zone of long T2 signal surrounding. The analysis with confirmation by pathological examination showed that the inner zone with equal T1 short T2 signal was a coagulated necrosis area, and the outer zone with long T2 signal showed pulmonary heat injury area including neutrophil infiltration, alveolar effusion, and pulmonary congestion. In this study, short T1 and short T2 signals were observed in the center of the thermal ablated zone on MRI. The maximum diameters of the central short T2 signal area and the central short T1 signal area after ablation are similar to those of the pathological coagulated necrosis area, which included tumor coagulated necrosis and lung coagulated necrosis area. Meanwhile, the maximum diameter of the pathological coagulated necrosis area was larger than that of the tumor before ablation but smaller than the maximum diameter of the GGO on the CT after ablation. The peripheral equal T1 long T2 signal area on MRI was considered to correspond to the pathological peripheral hemorrhage area and inflammatory layer area, both of which were larger than the peripheral GGO range on CT after ablation. It was indicated that MRI was more sensitive to the changes of intra-alveolar exudation than CT.

In this study, there was 1 case of needle tract bleeding during puncture in the RFA group, which affected the judgment of CT curative effect. Immediate post-ablation MRI could clearly distinguish the coagulation necrosis area from the bleeding area according to the characteristic changes, which showed a more objective evaluation of the ablation curative effect.

According to reports, MWA is less affected by the heat sink effect compared to RFA (14, 15). In this study, all 6 cases of rabbit lung VX2 tumors in the MWA group showed complete ablation by pathology. In the RFA group, 1 case showed residual tumor after ablation, which was located next to the blood vessel in the right lower lobe. Residual tumor was displayed as slightly equal T1 long T2 signal and considered to be related to the heat sink effect.

This study had certain limitations. First, the sample size of experimental animals was small. Second, contrast-enhanced MRI and CT scans were not used to further evaluate the efficacy of ablation. Third, only acute thermal ablation injury was observed immediately without dynamic observation for imaging and pathological evolution of ablated lesions. The shortcomings need to be improved in future research.

In conclusion, MRI manifestations of thermal ablation zones for rabbit lung VX2 tumors have certain characteristics and better corresponded with pathology than CT. CT combined with MRI multimodal radiomics is expected to provide an effective new method for clinical evaluation of the immediate efficacy of thermal ablation of lung tumors and to improve the complete tumor ablation rate.

## Data availability statement

The raw data supporting the conclusions of this article will be made available by the authors, without undue reservation.

## Ethics Statement

The animal study was reviewed and approved by First Affiliated Hospital of Fujian Medical University.

## Author contributions

ZL conceptualized the study, prepared figures and tables. JinC wrote the article and prepared figures and tables. YY collected the data, carried out the analysis, and prepared the figures and tables. QL, JiaC, and JieC participated in drafting and editing the article and assisted in the preparation of figures and tables. All authors contributed to the article and approved the submitted version.

## Funding

This study was funded by Develop Program of Focus Field of Guangdong Province, PRC (2019B110233001).

## Conflict of Interest

The authors declare that the research was conducted in the absence of any commercial or financial relationships that could be construed as a potential conflict of interest.

## Publisher’s note

All claims expressed in this article are solely those of the authors and do not necessarily represent those of their affiliated organizations, or those of the publisher, the editors and the reviewers. Any product that may be evaluated in this article, or claim that may be made by its manufacturer, is not guaranteed or endorsed by the publisher.
